# *U2AF1 S34F* enhances tumorigenic potential of lung cells by exhibiting synergy with *KRAS* mutation and altering response to environmental stress

**DOI:** 10.1101/2024.09.11.612492

**Published:** 2024-09-15

**Authors:** Cindy E Liang, Eva Hrabeta-Robinson, Amit Behera, Carlos Arevalo, Isobel J. Fetter, Cameron M. Soulette, Alexis M. Thornton, Shaheen S. Sikandar, Angela N. Brooks

**Affiliations:** 1 Department of Molecular, Cell and Developmental Biology, University of California, Santa Cruz, Santa Cruz, CA 95064, USA; 2 Department of Biomolecular Engineering, UC Santa Cruz, Santa Cruz, CA 95064, USA; 3 Co-first author

**Keywords:** RNA Sequencing, gene expression, splicing, cancer, U2AF1, KRAS, stress granule, proliferation, lung adenocarcinoma, genetic interactions

## Abstract

Although *U2AF1*^*S34F*^ is a recurrent splicing factor mutation in lung adenocarcinoma (ADC), *U2AF1*^*S34F*^ alone is insufficient for producing tumors in previous models. Because lung ADCs with *U2AF1*^*S34F*^ frequently have co-occurring *KRAS* mutations and smoking histories, we hypothesized that tumor-forming potential arises from *U2AF1*^*S34F*^ interacting with oncogenic *KRAS* and environmental stress. To elucidate the effect of *U2AF1*^*S34F*^ co-occurring with a second mutation, we generated human bronchial epithelial cells (HBEC3kt) with co-occurring *U2AF1*^*S34F*^ and *KRAS*^*G12V*^. Transcriptome analysis revealed that co-occurring *U2AF1*^*S34F*^ and *KRAS*^*G12V*^ differentially impacts inflammatory, cell cycle, and KRAS pathways. Subsequent phenotyping found associated suppressed cytokine production, increased proliferation, anchorage-independent growth, and tumors in mouse xenografts. Interestingly, HBEC3kts harboring only *U2AF1*^*S34F*^ display increased splicing in stress granule protein genes and viability in cigarette smoke concentrate. Our results suggest that *U2AF1*^*S34F*^ may potentiate transformation by granting precancerous cells survival advantage in environmental stress, permitting accumulation of additional mutations like *KRAS*^*G12V*^, which synergize with *U2AF1*^*S34F*^ to transform the cell.

## INTRODUCTION

Splicing factor mutations are prevalent in cancer and lead to global dysregulation of RNA splicing in protein-coding genes^1,2,3,4,5,6,7,8,9,10^. Work still remains to fully characterize the functional consequences of dysregulated splicing. *U2AF1* is among the most significantly mutated genes in lung ADC and codes for a splicing factor^11^. Of the *U2AF1* mutations in lung ADC, *U2AF1*^*S34F*^ occurs the most frequently^2^. U2AF1 is a subunit of the U2 Auxiliary Factor complex^12^. Wild-type U2AF1 (U2AF1^WT^) facilitates spliceosome assembly by recognizing and binding to the 3’ splice site^13^. U2AF1 can also directly bind to mRNA to repress protein translation^14^. In *U2AF1*^*S34F*^ cells, the amino acid substitution in the second zinc finger alters 3’ splice site choice^14,15,16^ and creates isoforms which result in abnormal gene expression^17^. Other impacts of this mutation include altered binding to mRNA leading to translational dysregulation, increased R loop formation, and reduced NMD activity^14,18,19,20^. Multiple functional consequences of this mutation have been reported, including increased survival advantage in cells exposed to ionizing radiation, altered inflammatory cytokine secretion, and increased stress granule production^14,21^. Despite these phenotypes, *U2AF1*^*S34F*^ by itself is insufficient for human lung cell lines (HBEC3kts) to form tumors in mouse xenograft experiments^15^. Confoundingly, *U2AF1*^*S34F*^ is associated with poorer prognosis in cancer patients, but the mutation decreases proliferation in cancer cell lines^22^. In lung ADC, *U2AF1*^*S34F*^ has been found to be a basal mutation^16,23^, indicating that it may potentiate the accumulation of further genetic perturbations under certain conditions or environments. Additionally, it has been observed to co-occur with known cancer-driver mutations like those in *KRAS* or *ROS1* fusions^11,16,23,24^. As such, understanding the impact of mutant *U2AF1* on oncogenesis in the context of different environments or with co-occurring mutations may pave the way for earlier diagnostic and treatment options for patients.

Although it has been hypothesized that *U2AF1*^*S34F*^ confers tumorigenic potential independent of that conferred by the driver mutations with which it co-occurs^11^, work still remains to fully understand U2AF1’s functional significance. Mutations in *KRAS* associated with lung cancer, alone, are also insufficient to independently cause *in vivo* transformation in HBEC3kts^25^. *KRAS* mutations have recently been reported to alter splicing through downregulating splicing factor phosphorylation^26^. The cooperativity of splicing perturbations caused by co-occurring *U2AF1*^*S34F*^ and *KRAS*^*G12V*^ in a pre-cancerous model has yet to be studied.

Here, we introduced *KRAS*^*G12V*^ to HBEC3kt lines with *U2AF1*^*S34F*^. We pair short-read mRNA sequencing with *in vivo* and *in vitro* assays to assess the impact of transcriptome alterations caused by co-occurring *U2AF1*^*S34F*^ and *KRAS*^*G12V*^ on preneoplastic potential and compare these perturbations to those caused by *U2AF1*^*S34F*^ or *KRAS*^*G12V*^ alone. Our results reveal synergistic effects of co-occurring *U2AF1*^*S34F*^ and *KRAS*^*G12V*^ in gene expression and splicing, which translate to enhanced oncogenic potential. Additionally, we discovered increased splicing in stress granule protein genes conferred by *U2AF1*^*S34F*^ alone, which is associated with enhanced resistance to environmental stress. We propose an evidence-based model for lung cancer oncogenesis in which *U2AF1*^*S34F*^ enhances preneoplastic potential by allowing cells to survive stress and synergize with the transcriptomic effects of subsequent accumulated mutations.

## RESULTS

### *KRAS*^*G12V*^ suppresses the effect of *U2AF1*^*S34F*^ on the transcriptome while altering gene expression in oncogenic pathways

**Figure 1:**
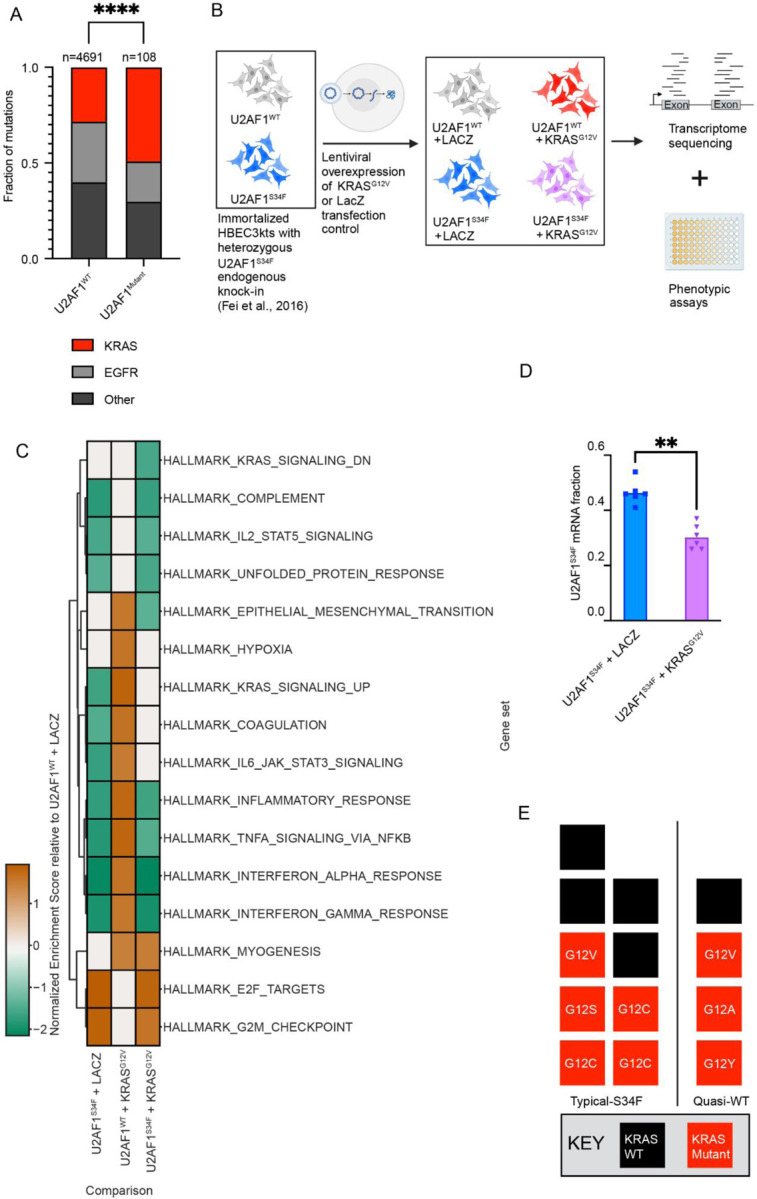
*KRAS*^*G12V*^ suppresses the effect of *U2AF1*^*S34F*^ on the transcriptome while altering gene expression in oncogenic pathways. **(A)** Distribution of *KRAS*, *EGFR*, and other mutations in lung ADC patients with and without mutations in *U2AF1*. (**B)** Experimental pipeline for study. Illumina RNA sequencing was performed on HBEC3kt lines with *U2AF1*^*S34F*^ alone, *KRAS*^*G12V*^ alone, co-occurring *U2AF1*^*S34F*^ and *KRAS*^*G12V*^, and a wild-type control. Phenotypic assays for oncogenic phenotypes were also performed. (**C)** Heatmap of gene enrichment scores for gene sets differentially expressed between each genotype and the wild-type control. (**D)** *U2AF1*^*S34F*^ mRNA fraction in HBEC3kt lines with differing mutational backgrounds. Bars represent mean *U2AF1*^*S34F*^ mRNA fraction. (**E)** Distribution of *KRAS* mutations in lung ADC patients observed to display quasi-WT or typical-S34F expression patterns. Each box represents a single patient. ** P ≤ 0.01, **** P ≤ 0.0001. See also Figures S1–S2, Table S1, Table S2.

To understand the role of *U2AF1*^*S34F*^ in cancer, we analyzed currently available sequencing data from lung ADC primary samples with *U2AF1* mutations to identify co-occurring mutations in known lung ADC driver genes. We find that *U2AF1*^*S34F*^ significantly co-occurs with *KRAS* mutations (Figure 1A, Table S1), with *KRAS* mutations at the *G12* locus being the most common. From this analysis, we identified *KRAS*^*G12X*^ as a candidate mutation to study in the context of *U2AF1*^*S34F*^.

We obtained two parental isogenic HBEC3kt clones that were either wild-type or mutant for *U2AF1*^15^: one cell line was homozygous *U2AF1*^*WT*^, and the other cell line was heterozygous for *U2AF1*^*S34F*^ at its endogenous locus (Figure S1 A). Homozygous *U2AF1*^*S34F*^ mutation is lethal, so was not a consideration^27^. A *KRAS*^*G12V*^ pLenti6_V5 plasmid^25^ has previously been used to identify genetic perturbations required to accomplish *in vivo* transformation of HBEC3kts, which we obtained to study the impact of co-occurring *U2AF1*^*S34F*^ and *KRAS*^*G12V*^ mutations on preneoplastic potential. We exogenously overexpressed *KRAS*^*G12V*^ in *U2AF1*^*WT*^ and *U2AF1*^*S34F*^ HBEC3kt cells using this construct. As a transduction control, we also introduced *LacZ* using the same plasmid backbone. A total of 4 cell lines were generated per parental HBEC3kt clone: (1) *U2AF1*^*WT*^ + *LacZ* (2) *U2AF1*^*S34F*^ + *LacZ,* (3) *U2AF1*^*WT*^ + *KRAS*^*G12V*^, (4) *U2AF1*^*S34F*^ + *KRAS*^*G12V*^. Lentivirus is known to unpredictably integrate into the host genome, and other groups’ use of this *KRAS*^*G12V*^ vector reported variation in *KRAS*^*G12V*^ expression^25^. The presence of *U2AF1*^*S34F*^ and *KRAS*^*G12V*^ was validated through transcript expression and immunoblot (Figures S1 B–F). Our immunoblot and gene expression analysis revealed that *KRAS* overexpression was inconsistent across the cell lines in clone 1 (Figures S2 A–E).

Using these four cell lines, we performed short-read RNA sequencing and cell-based assays to understand how *U2AF1*^*S34F*^ and *KRAS*^*G12V*^ co-occurrence alters the transcriptome and biology of HBEC3kt (Figure 1B). We performed differential gene expression and gene set enrichment analysis on RNA-seq data (Figure 1C, Table S2). A gene set uniquely downregulated in *U2AF1*^*S34F*^ + *KRAS*^*G12V*^ HBEC3kts is the KRAS Signaling Down gene set, corresponding to genes downregulated when KRAS is active^28^. In contrast, the *U2AF1*^*WT*^ + *KRAS*^*G12V*^ cell line had a positive enrichment for the KRAS Signaling Up gene set, corresponding to genes upregulated when KRAS is active, and no significant enrichment in the KRAS Signaling Down gene set. From this result, we infer that *U2AF1*^*S34F*^ in the presence of oncogenic *KRAS* alters the KRAS signaling pathway.

We also observed that individually, *U2AF1*^*S34F*^ and *KRAS*^*G12V*^ produced opposite enrichment patterns to each other in KRAS signaling, coagulation, and inflammatory pathway gene sets (KRAS Signaling Up, Coagulation, IL6 JAK STAT3 Signaling, Inflammatory Response, TNFA Signaling Via NFKB, Interferon Alpha Response, Interferon Gamma Response). In some gene sets (Complement, IL2 STAT5 Signaling, Unfolded Protein Response, Inflammatory Response, TNFA Signaling Via NFKB, Interferon Alpha Response, Interferon Gamma Response, E2F Targets, and G2M Checkpoint), the *U2AF1*^*S34F*^ enrichment pattern persisted in the *U2AF1*^*S34F*^ + *KRAS*^*G12V*^ cell line. In others (KRAS Signaling Up, Coagulation, IL6 JAK STAT3 Signaling), the opposite enrichment patterns caused by either mutation alone appeared to counter each other in the *U2AF1*^*S34F*^ + *KRAS*^*G12V*^ line, producing nonsignificant enrichment in the gene sets. This suggests that *KRAS*^*G12V*^ suppresses *U2AF1*^*S34F*^-specific gene expression signatures in the *U2AF1*^*S34F*^ + *KRAS*^*G12V*^ cell line. To understand this observation in the context of expression patterns in patient-derived samples, we quantified the ratio of *U2AF1*^*S34F*^ mRNA in cells with and without *KRAS*^*G12V*^ using our short-read RNA-seq data. A subset of lung ADC primary samples have been reported to have “quasi-WT” status, which represents tumors with low S34F:WT mRNA ratios, but unchanged absolute *U2AF1*^*S34F*^ or total *U2AF1* mRNA levels^15^. These quasi-WT S34F:WT mRNA ratios range from 0.27–0.31. We found that the *U2AF1*^*S34F*^ mRNA fraction in *U2AF1*^*S34F*^ + *KRAS*^*G12V*^ cells falls within the range of 0.26–0.37 (Figure 1D, Table S1) compared to the range of 0.41–0.54 when *U2AF1*^*S34F*^ is present alone. This suggests that the presence of *KRAS*^*G12V*^ may suppress *U2AF1*^*S34F*^ expression to the levels seen in “quasi-WT” patient samples.

We then examined the mutational status of typical-S34F and quasi-WT samples from The Camcer Genome Atlas (TCGA) lung ADC cohort studied by Fei et al.^15^. Consistent with our hypothesis that mutant *KRAS* suppresses *U2AF1*^*S34F*^ expression signature, we found that quasi-WT patient samples carry a higher proportion of *KRAS* mutations (3/4 samples) than typical-S34F samples (5/9) (Figure 1E), although the difference is not statistically significant, likely due to low sample sizes. Together, these results support the hypothesis that *KRAS*^*G12V*^ suppresses the *U2AF1*^*S34F*^ transcriptomic signature.

We also found variation in the *U2AF1*^*S34F*^ mRNA ratios between isogenic clones (Figure S2 F, Table S1). The *U2AF1*^*S34F*^ mRNA fraction of clone 1 *U2AF1*^*S34F*^ + *KRAS*^*G12V*^ ranged from 0.31–0.37, while the *U2AF1*^*S34F*^ mRNA fraction of clone 2 *U2AF1*^*S34F*^ + *KRAS*^*G12V*^ ranged from 0.26–0.28. Although clone 1 ranged higher in *U2AF1*^*S34F*^ mRNA ratio than clone 2, it was still well within the typical-S34F range reported by Fei et al. (0.43 or above)^15^. This result, along with our immunoblot of *KRAS*^*G12V*^ and KRAS gene expression analysis, indicated that inconsistent *KRAS*^*G12V*^ integration in clone 1 impacts both protein abundance and the transcriptome. Additionally, oncogenic KRAS has been shown to alter splicing^26^. Thus, we continued subsequent transcriptomic analyses and the majority of our phenotypic experiments interrogating the interaction of *U2AF1*^*S34F*^ and *KRAS*^*G12V*^ with clone 2.

### Co-occurring *U2AF1*^*S34F*^ and *KRAS*^*G12V*^ produces unique splicing event distributions, while *U2AF1*^*S34F*^ alone increases splicing in stress granule protein genes

Although *U2AF1*^*S34F*^ and oncogenic *KRAS* are both known to alter splicing^2,3,14,15,16,17,26,29,30,31^, the effect of co-occurring *U2AF1*^*S34F*^ and *KRAS*^*G12V*^ on differential splicing is unknown. First, we examined the mutations’ global effects on splicing.

**Figure 2:**
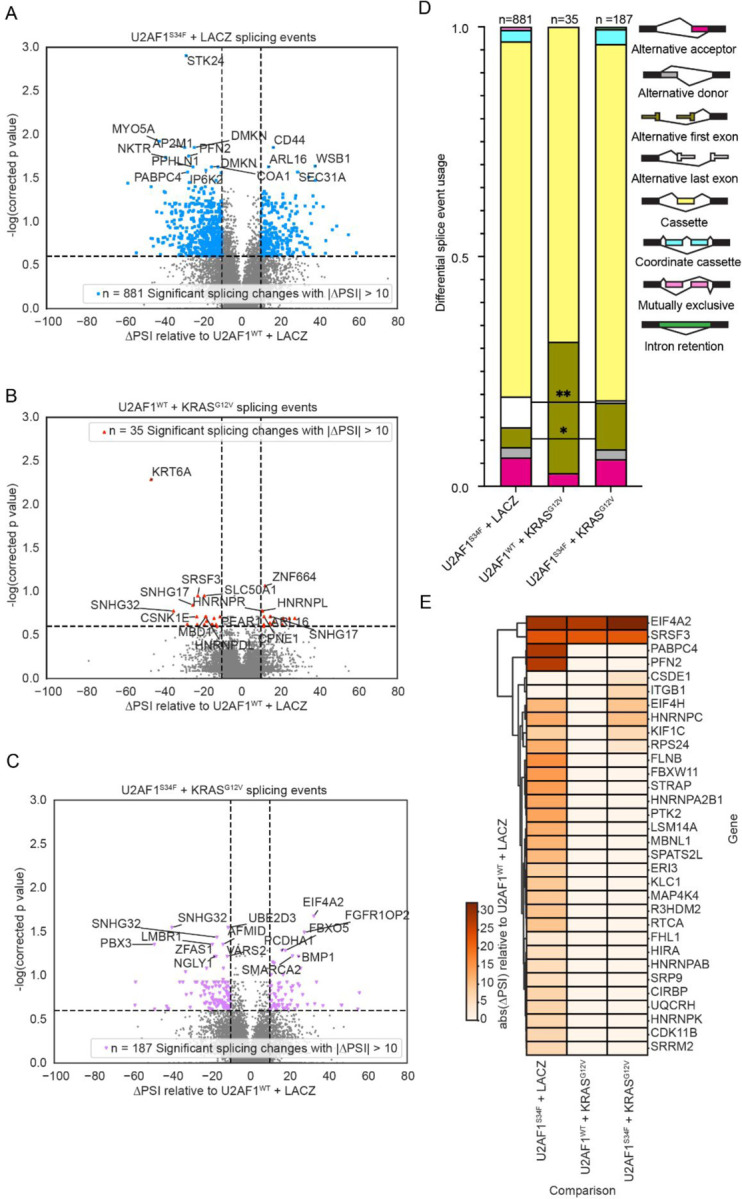
Co-occurring *U2AF1*^*S34F*^ and *KRAS*^*G12V*^ produces unique splicing events distributions, while *U2AF1*^*S34F*^, alone, increases splicing in stress granule protein genes. **(A-C)** Volcano plots of differentially spliced genes compared to the wild-type control in *U2AF1*^*S34F*^ + *LacZ*, *U2AF1*^*WT*^ + *KRAS*^*G12V*^, and *U2AF1*^*S34F*^ + *KRAS*^*G12V*^ HBEC3kts. Colored dots and numbers displayed in inset represent genes with significant splicing changes (adjusted p < 0.25) of magnitude greater than 10% ( |ΔPSI| ≥ 10). The top 15 most significantly differentially spliced genes were labeled. (**D)** Distribution of splicing event types as categorized by JuncBASE (**E)** Heatmap of splicing changes in stress granule protein genes. * P ≤ 0.05, ** P ≤ 0.01. See also Figure S3A, Table S1, Data S1.

We detected and quantified splicing events from short-read data using JuncBASE^32^ (See Data Availability). As expected, *U2AF1*^*S34F*^ + *LacZ* HBEC3kts exhibited the most changes in differentially spliced genes, compared to *U2AF1*^*WT*^ + *LacZ* (Figure 2A). *U2AF1*^*WT*^ + *KRAS*^*G12V*^ HBEC3kts displayed the lowest amount of differentially spliced genes (Figure 2B), while *U2AF1*^*S34F*^ + *KRAS*^*G12V*^ HBEC3kts displayed an intermediate number (Figure 2C). Consistent with the effect that *KRAS*^*G12V*^ has on expression of *U2AF1*^*S34F*^, these results suggest that *KRAS*^*G12V*^ suppresses the splicing changes mediated by *U2AF1*^*S34F*^.

We next compared the categories of splicing events that were significantly different (adjusted p < 0.25 and |ΔPSI| ≥ 10) between *U2AF1*^*S34F*^ + *LacZ* and *U2AF1*^*WT*^ + *LacZ*, and *U2AF1*^*S34F*^ + *KRAS*^*G12V*^ and *U2AF1*^*WT*^ + *LacZ*. JuncBASE categorizes splicing events in eight different categories: cassette exon, mutually exclusive exon, coordinate cassette exons, alternative 5’ splice site, alternative 3’ splice site, alternative first exon, alternative last exon, and retained intron (Figure 2D, Table S1). We found that co-occurring *U2AF1*^*S34F*^ and *KRAS*^*G12V*^ mutations had a similar fraction of cassette exon events as *U2AF1*^*S34F*^ alone, a splicing event type characteristic of *U2AF1*^*S34F* 2,14,15,17,30,31^. This persistence of *U2AF1*^*S34F*^-specific splicing event was consistent with our gene expression results, which demonstrated that certain *U2AF1*^*S34F*^-specific enrichment patterns persisted in the *U2AF1*^*S34F*^ + *KRAS*^*G12V*^ line. In contrast, the alternative first exon and alternative last exon events in *U2AF1*^*S34F*^ + *KRAS*^*G12V*^ HBEC3kts appeared to be at an intermediate fraction between those in *U2AF1*^*S34F*^ + *LacZ* and *U2AF1*^*WT*^ + *KRAS*^*G12V*^ cells. Similar to gene expression enrichment patterns, we hypothesize that the effects of co-occurring *U2AF1*^*S34F*^ and *KRAS*^*G12V*^ mutations on splicing antagonize with each other to create intermediate splicing events proportions.

To examine potential biological pathways impacted by the differentially spliced genes, we performed gene set enrichment analysis (GSEA) on differentially spliced genes. In contrast to the differential gene expression GSEA results, far fewer Hallmark gene sets were significantly differentially enriched amongst our genotypes. Only one gene set, p53 Pathway, was found to be significantly enriched, and only in the *U2AF1*^*S34F*^ + *KRAS*^*G12V*^ comparison. This finding highlights the non-overlapping roles of gene expression and splicing on transcripts belonging to certain pathways in the cell.

A recently appreciated role of *U2AF1*^*S34F*^ is its ability to confer resistance to the effects of stress. *U2AF1*^*S34F*^ alone has been shown to increase cell proliferation following ionizing radiation exposure^14^. *U2AF1*^*S34F*^ has also recently been found to confer altered splicing and binding to stress granule gene sets in an MDS cell line^21^. Stress granules are RNA-protein condensates that may help cells survive stress and can help cancer cells resist chemotherapy^33^. In the MDS line, altered splicing in stress granule genes was associated with enhanced viability under sodium arsenite, a chemical agent of oxidative stress^21^.

We hypothesized *U2AF1*^*S34F*^ may also be altering stress response through aberrant splicing in our cell lines. We observed increased splicing in stress granule protein genes in *U2AF1*^*S34F*^ + *LacZ*, compared to other genotypes (Figure 2E, Data S1) Interestingly, gene expression changes in in cells with *U2AF1*^*S34F*^ background also showed increased expression in this gene set compared to cell lines without *U2AF1* mutation (Figure S3 A, Table S2).

### Co-occurring *U2AF1*^*S34F*^ and *KRAS*^*G12V*^ mutations increase oncogenic potential and proliferation

**Figure 3:**
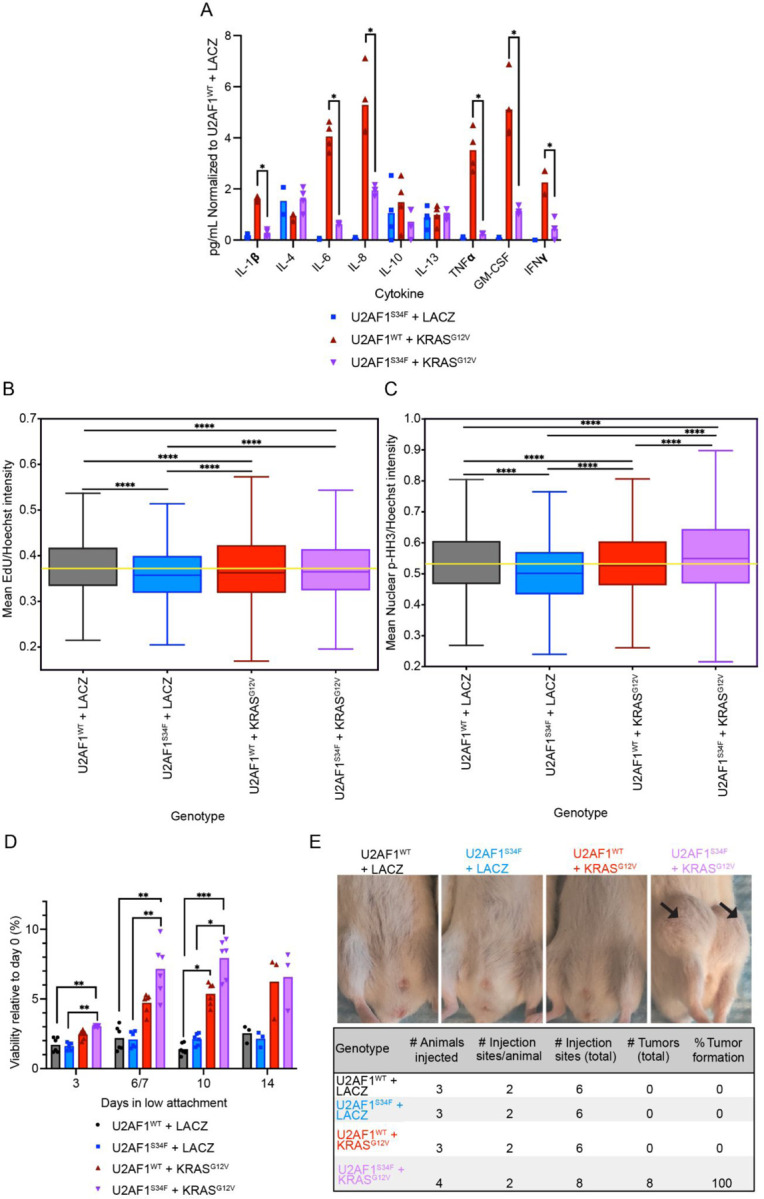
Co-occurring *U2AF1*^*S34F*^ and *KRAS*^*G12V*^ mutations increase oncogenic potential and proliferation. **(A)** Secreted cytokine measurements of each genotype, normalized to *U2AF1*^*WT*^ + *LACZ* levels. Bars represent mean normalized cytokine concentration. (**B)** Mean EdU intensity of cells, normalized by cell density. (**C)** Mean PHH3 intensity, normalized by cell density. Yellow lines correspond to the median of the wild-type control. The middle line in the body of each boxplot represent medians of each genotype, box limits represent quartiles, and whiskers represent the range of the most extreme, non-outlier data points. (**D)** Viability in low-attachment vessel for each HBEC3kt genotype. Relative viability is calculated by dividing the viability for each genotype at a certain time point, by the genotype’s viability at day 0. Bars represent mean viability. (**E)** Top, representative injection site images of mouse xenografts. Bottom, tumor formation quantification for each HBEC3kt genotype injected. * P ≤ 0.05, ** P ≤ 0.01, *** P ≤ 0.001, **** P ≤ 0.0001. See also Figure S3B–E, Table S1, Table S3.

Following this transcriptomic profiling, we sought to understand the functional consequences of differentially expressed gene sets. We first sought to explore gene sets with similar enrichment patterns in both *U2AF1*^*S34F*^ + *LacZ* and *U2AF1*^*S34F*^ + *KRAS*^*G12V*^ HBEC3kts, as they indicated *U2AF1*^*S34F*^-specific effects which persisted when *KRAS*^*G12V*^ was present. One category that fit this criteria was the inflammatory pathway gene sets (Complement, IL2 STAT5 Signaling, IL6 JAK STAT3 Signaling, Inflammatory Response, TNFA Signaling Via NFKB, Interferon Alpha Response, and Interferon Gamma Response), where the presence of *U2AF1*^*S34F*^ was associated with downregulation. Oncogenic Ras has been found to increase production of cytokines such as IL-6 in multiple cell types^34,35^. To probe how these pathways are altered in our *U2AF1*^*S34F*^ + *KRAS*^*G12V*^ cell line, we measured inflammatory cytokine production in our HBEC3kt cell lines. For most cytokines tested, we observe that *U2AF1*^*WT*^ + *KRAS*^*G12V*^ HBEC3kts secrete the highest levels of inflammatory cytokines. Co-occurrence of *U2AF1*^*S34F*^ with *KRAS*^*G12V*^ suppresses the levels of secreted cytokines IL-1β, IL-6, IL-8, TNFα, GM-CSF, and IFNγ (Figure 3A, Table S1). High levels of IL-1β, TNFα, GM-CSF, and IFNγ have been shown to promote antitumor activity in animal models^36,37,38,39^. We hypothesized that *U2AF1*^*S34F*^ creates a microenvironment conducive to tumor growth by bringing cytokine secretion down to an intermediate level in *KRAS*^*G12V*^-mutant cells. We note that our cytokine results are inconsistent with previous work done on *U2AF1*^*S34F*^ HBEC3kts, which showed that *U2AF1*^*S34F*^ increases the secretion of cytokines such as IL-8^14^. However, the clonal background of the cells used in the aforementioned study was not reported, and it is possible that different steady-state cytokine secretion levels may be present in HBEC3kts from different isogenic clones.

HBEC3kts with *U2AF1*^*S34F*^ also exhibited expression in gene sets related to cell cycle progression (E2F Targets, G2M checkpoint). To understand how these gene expression differences translate to altered phenotype, we next asked how *U2AF1*^*S34F*^ and *KRAS*^*G12V*^ mutations affect proliferative potential. We stained HBEC3kts with EdU and phospho histone-H3 (PHH3) to measure the proportion of cells undergoing S-phase and M-phase, respectively^40,41^ (Figures 3B–C, Table S3). Previous models with *U2AF1*^*S34F*^ have found that *U2AF1*^*S34F*^ by itself suppresses growth phenotypes such as proliferation and colony-forming potential^15,22^. Consistent with these findings, we observed lower normalized EdU and PHH3 intensity in *U2AF1*^*S34F*^ + *LacZ* HBEC3kts compared to *U2AF1*^*WT*^ + *LacZ*. However, when *KRAS*^*G12V*^ and *U2AF1*^*S34F*^ co-occur, we observe increased proliferation compared to *U2AF1*^*S34F*^ by itself. Notably, M-phase staining in *U2AF1*^*S34F*^ + *KRAS*^*G12V*^ HBEC3kts was elevated to above *U2AF1*^*WT*^ + *LacZ* levels (Figure 3C), indicating that *KRAS*^*G12V*^ confers increased mitosis in *U2AF1*^*S34F*^-mutant cells.

Mutant *KRAS*, including *KRAS*^*G12V*^ is known to induce oncogenic phenotypes, such as anchorage-independent growth^42^. Due to the enhanced proliferation in *U2AF1*^*S34F*^ + *KRAS*^*G12V*^ cells and the unique negative enrichment score in the KRAS Signaling Down gene set observed in this line, we hypothesized that co-occurring *U2AF1*^*S34F*^ and *KRAS*^*G12V*^ would alter anchorage-independent growth as well. We cultured HBEC3kts of the four genotypes on low attachment plates and measured viability. *U2AF1*^*S34F*^ + *KRAS*^*G12V*^ HBEC3kts survived anchorage-independent growth conditions better than other genotypes over 10 days in low-attachment conditions (Figure 3D, Table S1).

Finally, we sought to understand how *U2AF1*^*S34F*^ and *KRAS*^*G12V*^ co-occurrence in HBEC3kts impacts the ability of cells to form tumors, *in vivo*. Cells from the four genotypes were injected into NOD scid gamma (NSG) immunodeficient mice. We find that *U2AF1*^*S34F*^ + *KRAS*^*G12V*^ HBEC3kts formed more tumors than HBEC3kts with either mutation alone (Figure 3E). This suggests that co-occurring *U2AF1*^*S34F*^ and *KRAS*^*G12V*^ mutations synergize to transform HBEC3kts cells *in vivo*.

Similar to the heterogeneity observed in *KRAS* gene expression, we also observed phenotypic heterogeneity between our isogenic clones. When we asked how the tumor formation differed between cells from clone 1 and clone 2, we found that clone 2 *U2AF1*^*S34F*^ + *KRAS*^*G12V*^ HBEC3kts formed tumors more frequently than clone 1 (Figure S3 B). In contrast, the one tumor formed by a *U2AF1*^*WT*^ + *KRAS*^*G12V*^ was from clone 1. Similarly, when we examined viability in low-attachment conditions, we observed that the *U2AF1*^*S34F*^ + *KRAS*^*G12V*^ cell line from clone 2 was more viable in low attachment conditions at earlier timepoints than clone 1 (Figure S3 C, Table S1). Importantly, no tumors were formed with *U2AF1*^*S34F*^, suggesting that *U2AF1*^*S34F*^ alone is insufficient for *in vivo* transformation. These results are consistent with the hypothesis that inconsistent *KRAS*^*G12V*^ integration in clone 1 impacts oncogenic potential unpredictably.

We also assayed for other cancer hallmarks in clone 2, such as the long-term ability to survive and proliferate into colonies, which is a marker of cancer stemness and can be assessed with a clonogenicity or colony-forming assay^43^. Interestingly, co-occurring *KRAS*^*G12V*^ and *U2AF1*^*S34F*^ suppressed colony-forming potential (Figure S3 D, Table S1). These findings are consistent with previous work performed on *U2AF1*^*S34F*^-mutant cancer cell lines^22^. We also performed a wound-healing assay to assess the invasive potential of *U2AF1*^*S34F*^ + *KRAS*^*G12V*^ HBEC3kts (Figure S3 E, Table S1) and observed that *U2AF1*^*S34F*^ decreases invasive potential in *KRAS*^*G12V*^ background. Our results indicate that enhanced proliferation conferred by *U2AF1*^*S34F*^ + *KRAS*^*G12V*^ may work in concert with pathways outside of stemness and invasion to confer oncogenic potential.

### Altered splicing in stress granule genes in *U2AF1*^*S34F*^ HBEC3kts is associated with enhanced stress response

**Figure 4:**
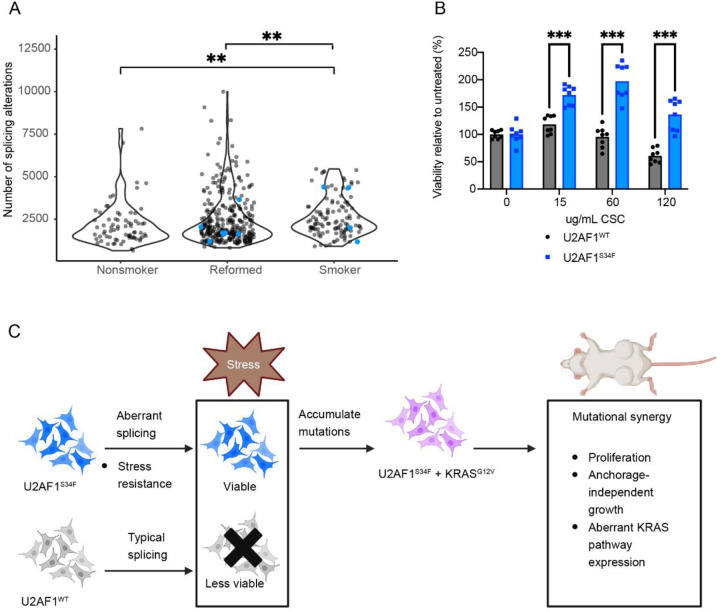
Altered splicing in stress granule genes in *U2AF1*^*S34F*^ HBEC3kts is associated with enhanced stress response. **(A)** Splicing alteration distribution in lung ADC patients with smoking histories. Blue dots represent patients with *U2AF1* mutations. (**B)** Viability in cigarette smoke concentrate (CSC). Concentrations are in ug/mL CSC. Bars show mean viability of each cell line. **(C)** Working model for *U2AF1*^*S34F*^’s role in priming cells for oncogenic transformation. ** P ≤ 0.01, *** P ≤ 0.001. See also Data S2, Table S1.

Stress granules are often induced by an agent of oxidative stress^21,44^. Oxidative stress is relevant to cancer formation because it can produce mutations by causing DNA damage^45^. In lung ADC patients, a common source of oxidative stress is exposure to cigarette smoke. To understand the connection between oxidative stress response and lung ADC, we next analyzed splicing alterations in primary sample data from TCGA. We found greater numbers of splicing alterations in lung ADC samples from patients with smoking histories than in never-smokers (Figure 4A and See Data Availability). Although we observed a trend of increased *U2AF1* mutations in samples from patients with smoking histories, high splicing alterations were present in *U2AF1*^*WT*^ samples in this group as well.

Previous studies on *U2AF1*^*S34F*^ have observed an increase in viability after exposing cells to oxidative stress like radiation and sodium arsenite, compared to *U2AF1*^*WT*^ cells^14,21^. We followed up on this line of inquiry by measuring how *U2AF1*^*S34F*^ alone impacts viability in cigarette smoke concentrate (CSC) (Figure 4B, Table S1). Because enhanced resistance to stress appeared to be conferred by U2AF1^S34F^ alone, We treated *U2AF1*^*S34F*^ or *U2AF1*^*WT*^ HBEC3kts from both clone 1 and clone 2 with CSC and measured viability after three days. *U2AF1*^*S34F*^ HBEC3kts displayed higher viability than *U2AF1*^*WT*^ HBEC3kts at all concentrations tested.

Together, our results lead us to a model of oncogenic transformation. *U2AF1*^*S34F*^ has been reported to be a truncal mutation in lung cancer and MDS^16,23,46^. We propose that *U2AF1*^*S34F*^, when present in precancerous cells, allows cells to survive an initial onslaught of oxidative stress better. The surviving cells are more likely to persist and accumulate further mutations like *KRAS*^*G12V*^, which act synergistically with *U2AF1*^*S34F*^ to alter splicing and gene expression, resulting in an increase in oncogenic potential (Figure 4C).

## DISCUSSION

Despite its status as a recurrent mutation, the role of *U2AF1*^*S34F*^ in lung cancer has been difficult to understand since the mutation confers anti-proliferative and anti-invasive phenotypes when present alone in model systems. This aspect limits the ability of researchers to identify the functional role of *U2AF1*^*S34F*^ in lung cancer and limits the use of *U2AF1*^*S34F*^ as a prognostic marker for lung ADC. To gain a better understanding of this mutation, we examined the role of *U2AF1*^*S34F*^ in early cancer formation in two directions: how *U2AF1*^*S34F*^ may synergize with other cancer drivers like *KRAS*^*G12V*^, and how *U2AF1*^*S34F*^ by itself can impact stress response in the cell.

Validation of pathways predicted to be differentially altered by gene set enrichment analysis revealed that the direction of enrichment in a gene set did not always correspond with the direction of pathway change in phenotype. For instance, although cell cycle gene sets were both positively enriched in *U2AF1*-mutant cell lines regardless of *KRAS* status, *U2AF1*^*S34F*^ + *LacZ* cells exhibited reduced proliferation. In contrast, the co-occurrence of *U2AF1* and *KRAS* mutations increased proliferation. Other gene set enrichment patterns translated to more consistent phenotypes. For instance, a negative enrichment in inflammatory gene sets translated to suppression of inflammatory cytokines for HBEC3kt lines.

We also examined splicing-level changes in the transcriptome caused by *U2AF1* and *KRAS* mutations. Interestingly, we found little overlap in gene set enrichment between differentially expressed and differentially spliced genes, highlighting the importance of using multiple kinds of RNA-seq analysis to assess the synergistic impact of mutations. For example, we observed increased alteration in stress granule protein genes unique to samples harboring *U2AF1*^*S34F*^ in both splicing analysis and differential gene expression results. We also detected *U2AF1*^*S34F*^-specific splicing event types, such as cassette exon event usage, that persisted in *U2AF1*^*S34F*^ + *KRAS*^*G12V*^ cells, while *KRAS*^*G12V*^ presence suppressed other *U2AF1*^*S34F*^-specific event types to an intermediate level.

When we followed up on our splicing analysis by quantifying cellular stress response, we found that *U2AF1*^*S34F*^ confers resistance to exposure to cigarette smoke concentrate. Our work leads us to a model in which *U2AF1*^*S34F*^ confers oncogenic potential that is dependent on the presence of environmental stress. When stress is present, we propose that *U2AF1*^*S34F*^ confers a survival advantage over *U2AF1*^*WT*^ cells, which allows for continued proliferation and accumulation of stronger oncogenic drivers like *KRAS*^*G12V*^, which synergize with *U2AF1*^*S34F*^ to increase oncogenic potential.

More work is left to be done to understand the role of splicing in stress response. For instance, the persistence of cassette exon events in *U2AF1*^*S34F*^ + *KRAS*^*G12V*^ cells highlights this category of splicing events as an interesting candidate for further functional study. Intron retention (IR) is another splicing event type linked to stress response in eukaryotes. In yeast, IR has been linked to fitness advantage in the presence of environmental stressors like starvation^47^. In mouse cells, IR has been linked to osmotic stress^48^. Although we did not find evidence of altered intron retention in our short-read analysis, previous work from our group utilizing long-read sequence analysis has shown *U2AF1*^*S34F*^ increasing IR from long-read data^17^, leaving an interesting avenue to pursue as long-read sequencing technologies improve.

Overall, our study points to the importance of interrogating the function of cancer mutations in the context of other mutational contexts and environmental conditions to obtain a more complete understanding of their contributions to tumorigenesis.

### Lead contact

Please direct all manuscript questions to Dr. Angela Brooks (anbrooks@ucsc.edu)

### Material availability

Cell lines generated from this study are available upon request.

### Data and code availability

Sequence data have been deposited at GEO under GSE267349. JuncBASE splicing quantification of cell lines and primary TCGA samples are deposited in Zenodo: https://zenodo.org/records/12774112. All code have been deposited to GitHub is publically available at https://github.com/cindyeliang/u2af1-kras/tree/main/scripts.

## EXPERIMENTAL MODEL DETAILS

### Mouse models

*NOD.Cg-Prkdc*^*scid*^*Il2rg*^*tm1Wjl*^*/SzJ* (NSG) (stock #005557) mice were purchased from The Jackson Laboratory and bred at the University of California Santa Cruz (UCSC). All mice used for this study were maintained at the UCSC Animal Facility in accordance with the guidelines set forth by UCSC and the Institutional Animal Care and Use Committee (Protocol number SIKAS2010).

## METHOD DETAILS

### Cell lines:

Host HBEC3kt cell lines homozygous for wildtype *U2AF1* and heterozygous with one copy of *U2AF1*^S34F^ at the endogenous locus were obtained as a gift from the laboratory of Harold Varmus (Cancer Biology Section, Cancer Genetics Branch, National Human Genome Research Institute, Bethesda, United States of America and Department of Medicine, Meyer Cancer Center, Weill Cornell Medicine, New York, United States Of America) and maintained as described by Fei et al.^15^. These cell lines were used for lentiviral transduction and blasticidin selection to generate a stable expression of *KRAS*^G12V^ or *LACZ* using plasmids obtained as gifts from the laboratory of John D Minna (Hamon Center for Therapeutic Oncology Research, The University of Texas Southwestern Medical Center) and used as described in Sato et al.^25^. Cell lines generated were tested for mycoplasma (IDEXX).

### Mouse xenograft of HBEC3kts:

Clone 1 and clone 2 HBEC3kt lines were cultured as previously described^15^. Cells were allowed to recover from cold storage in liquid nitrogen after seeding for one passage in a T-25 flask. Cells were passaged to a 10cm plate, then to a final 15cm plate, and allowed to grow to 80% confluency. At 80% confluency, the media in the 15cm plates were aspirated, and cells were washed twice with DPBS. To suspend cells for injection, cells were trypsinized with standard protocols^49^, and live cell counts were assessed by Trypan Blue staining. For each cell line, 9 million cells were resuspended in Keratinocyte SFM media containing 40% Matrigel and subcutaneously injected into the fourth abdominal fat pads on both sides of male NSG mice. 2–5 million cells were injected at each site in 100 uL media + Matrigel (5 million in 1st xenograft experiment, 2 million in 2nd). Mice were monitored every week for tumor growth. All mice were euthanized if tumor growth reached end point (1500 mm^3^), the tumors were ulcerated, or mice showed signs of distress. Tumor size was measured using digital calipers. A total of 4 female and 14 male mice were used.

### RNA extraction of HBEC3kts:

For RNA sequencing of HBEC3kt lines, cells were allowed to recover from cold storage in liquid nitrogen after seeding for one passage in a T-25 flask. Cells were passaged at 70–80% confluency to maintain log-phase growth into a 10cm plate. Once cells in the 10cm plates reached 70–80% confluency, they were washed twice in ice-cold DPBS and then collected in Tri-reagent for storage at −80°C until the bulk RNA was extracted using Direct-Zol RNA Miniprep Kit (Cat#R2050, Zymo Research).

### Short-read RNA-Seq of *U2AF1*^*WT*^ + *LACZ*, *U2AF1*^*S34F*^ + *LACZ*, *U2AF1*^*WT*^ + *KRAS*^*G12V*^, and *U2AF1*^*S34F*^ + *KRAS*^*G12V*^ HBEC3kts:

For Illumina sequencing, n=3 10cm plates per HBEC3kt genotype of both clones, for a combined n=6 per genotype, were cultured for RNA extraction as described above. Concentrations of purified RNA in nuclease-free water were determined by Nanodrop-2000 Spectrophotometer and Qubit RNA BR Assay (ThermoFisher Scientific). RINe numbers ranging from 7.8–10 were determined by TapeStation 4150 RNA ScreenTape Analysis (Agilent Technologies) before sending RNA to UC Davis DNA Technologies and Expression Analysis Core Laboratory for poly-A strand specific library preparation to obtain 60 million paired end read pairs by NovaSeq S4 (PE150) sequencing.

### Viability assay:

HBEC3kts of differing genotypes were seeded in a 96 well-plate in triplicate and grown in supplemented KSFM. At multiple time points, (0, 4, and 6 days), cells were rinsed twice with DPBS, CellTiter-Glo (Cat# PRG7572, Promega) reagent was added, and cells were transferred to white opaque 96 well-plates for luminescence measurement. Luminescence at each timepoint was quantified using the VarioSkan platereader (Thermo Scientific) and normalized to the average relative luminescence units (RLU) of the 0 day timepoint.

### Western blot analysis:

Cell lines were cultured to 85% confluency in 10cm plates. After preparation of protein lysates in 1ml of RIPA buffer supplemented with protease inhibitor cocktail (Cat# 5892970001, Roche Molecular Systems, Inc, USA) proteins were denatured using standard denaturation techniques in beta mercaptoethanol laemmli buffer, and 15ug of denatured protein lysate was separated on a 4–15% Mini-Protean TGX Precast Protein Gel (Cat# 4561086, Bio-Rad Laboratories, Inc. USA). After transfer to 0.2 um PVDF membrane using TransBlot Turbo Transfer system (Cat# 1704272, BioRad Laboratories), membranes were incubated shaking at room temperature in 5% milk block in 1x PBST followed by incubation in KRAS^G12V^ primary antibody at 1:250 dilution (Cat# 14412, Cell Signaling Technologies) and B actin conjugated to HRP at 1:500 dilution (Cat# sc-47778 HRP, Santa Cruz Biotechnology) in milk block overnight on an orbital shaker at 4°C.The next day, blots were washed in PBST and incubated with secondary HRP-conjugated antibody (Cat# 7074, Cell Signaling Technologies) at 1:1000 dilution at room temperature for 1h. After washing in PBST, bands were detected using WesternSure PREMIUM Chemiluminescent substrate (Cat# 926–95000, Li-COR Biosciences) and visualized on a C-Digit Blot Scanner (Li-COR Biosciences).

### Secreted cytokine analysis:

Growth triplicates of each cell line were seeded in 6 well plates and cultured with standard protocols described above to 85% confluency. Conditioned media (3 mL) above the cells was collected and cell debris spun out at 3000 x g for 10 mins at 4°C and supernatent was stored in −80°C before sending to Eve Technologies (Calgary, Canada) for the Human High Sensitivity T-Cell Discovery Array 14-plex (HDHSTC14) assay. Data was plotted and significance was calculated with a Mann-Whitney test on GraphPad Prism.

### Proliferation immunofluorescent assays:

Cell staining was performed at UCSC’s Chemical Screening Center, using the BioTek EL406 with peri/syringe/wash modules for automated washing and dispensing of reagents. Cells were cultured as previously described in optical-bottom black opaque 96 well-plates (Cat# 3904, Corning). The plate was taken to the Chemical Screening Center and incubated with EdU for 1 hour at 37°C and 5% CO2. Following EdU incorporation, cells were fixed with 5% formaldehyde (Cat# F79-500, Fisher) in basal media (Cat# PCS-300-030, ATCC) for 30 minutes at 37°C and 5% CO2. Cells were blocked with 2% BSA in PBST for 20–60min in the dark at room temperature. Following blocking, click reagent (15ml 100mM Tris pH7.4, 0.6ml 100mM CuSO4, 155.5ul 200mg/ml Na Ascorbate, 15.5ul 10mg/ml Rhodamine-Azide) was added to the cells to visualize EdU incorporation and cells were incubated in the dark for 1h at room temperature. Following azide incorporation, cells were stained with Hoechst (2.5uL in 2% BSA) to visualize nuclei and incubated in the dark for 2h at room temperature. Cells were then incubated with a primary antibody for PHH3 (Ser10) Recombinant Rabbit Monoclonal Antibody (9H12L10) (Cat# 701258, Invitrogen) at 1:5000 dilution in PBS and BSA, followed by incubation in a chicken anti-Rabbit IgG (H+L) Cross-Adsorbed Secondary Antibody, Alexa Fluor^™^ 647 secondary antibody (Cat# A-21443, Invitrogen) at 1:1000 dilution.

### Immunofluorescent imaging and analysis:

Imaging and quantification of EdU, Hoechst, and PHH3 immunofluorescent signal was performed with the Perkin Elmer Opera Phenix Plus and Harmony bioinformatics software at UCSC’s Chemical Screening Center. Valid cells were identified as nonborder objects with the presence of nuclear Hoechst staining. The mean EdU and PHH3 intensities of each valid object were divided by the mean Hoechst intensities to normalize for cell density. N = 12 wells in a 96 well-plate were seeded per genotype and at least 36,000 objects were analyzed per genotype. The resulting values were plotted and statistical analysis was performed on Python.v3.7.7 and Jupyter notebook v6.3.0. Significance was calculated using a Kruskal-Wallis test with Dunn’s multiple comparisons test.

### Growth in Low Attachment (GILA):

Cells were grown to 85% confluency on regular tissue culture-treated 6-well plates, harvested by trypsinization, filtered over a nylon 70um mesh and seeded in triplicate in KSFM media at 2500 cells per well in Ultra-low attachment 96 well plates (Cat# 3474, Corning) and time points were collected for viability assays over an 14-day period. An early time point was collected at the time of seeding and used for normalization. Viability was assayed using CellTiterGlo according to manufacturer’s instructions (Cat# G7570, Promega) and luminescence was measured on a VarioScan LUX plate reader (ThermoFisher). Significance was calculated using a Kruskal-Wallis test with Dunn’s multiple comparisons test using GraphPad Prism.

### Clonogenicity assay:

Colony formation was assessed by seeding the cells in triplicate at 200 cells per 10cm plate and cultured under normal conditions, except that media was changed only twice over a 10 day period so as not to disturb colony formation. Cells were fixed in 100% methanol for 20 mins and stained in 0.5% Crystal Violet in 25% methanol for 5 mins before drying and photographing. Colonies of approximately 2mm or larger were counted in 4 separate quadrants of each plate. Significance was calculated using a Kruskal-Wallis test with Dunn’s multiple comparisons test using GraphPad Prism.

### Wound healing assay:

HBEC3kts were seeded in 6-well plates. A 200uL pipettor and filter tip was used to create the wound in a confluent monolayer of cells. The wound was imaged at 0 and 3 hours. The number of cells that had migrated into the wound between the two time points was counted. Significance was calculated using a Kruskal-Wallis test with Dunn’s multiple comparisons test using GraphPad Prism.

### Cigarette smoke treatment:

CSC was obtained from Murty Pharmaceuticals (Cat# nc1560725). HBEC3kts from clone 1 and clone 2 were seeded in a 96 well-plate and grown to 50% confluency. Cells were then treated with 0, 15, 60, and 120ug/mL CSC for three days. Following treatment, the cells were washed twice with DPBS and assayed using CelTiterGlo as described above. Luminescence was normalized to the 0ug/mL control. For each genotype, data was combined from n = 4 of 2 clones, for a total n of 8. Significance was calculated using a Mann-Whitney test using GraphPad Prism.

### RNA-Seq Data Analysis:

Raw sequencing reads in fastq files were aligned to a version of the human genome hg38 that has a region of repeats masked to make the *U2AF1* locus alignable^50^, using STAR.v2.7.3a^51^ with the parameters --outSAMtype BAM SortedByCoordinate --twopassMode Basic --quantMode GeneCounts --bamRemoveDuplicatesType UniqueIdentical and the Gencode v33 primary assembly gtf file. Aligned BAM files were indexed with Samtools.v1.10^52^. Mapped reads in BAM files were counted with HTSeq.v0.12.4^53^ for all the annotated genes in gencode.v33.primary_assembly.annotation.gtf with -stranded = reverse and nonunique=none parameters.

### *U2AF1*^*S34F*^ mRNA ratio:

Aligned reads from clone 1 and clone 2 HBEC3kts were loaded onto the Integrative Genomics Viewer^54^ (IGV) at the *U2AF1*^*S34F*^ mutational locus. The fraction of A (mutant) nucleotides at this locus obtained from IGV was plotted. Significance was calculated with a Mann-Whitney test on GraphPad Prism.

### Differential expression analysis:

Differential expression analysis was performed with DESeq2 v1.40.2^55^ on R v4.3.1 on aligned RNA sequences from clone 1 and clone 2 of our HBEC3kt lines. Gene counts were normalized and a likelihood ratio test calculation was performed to account for batch differences between samples from clone 1 and clone 2. Statistical analysis was performed on expression differences in the following pairwise comparisons: *U2AF1*^*S34F*^ + *LacZ* vs. *U2AF1*^*WT*^ + *LacZ*, *U2AF1*^*WT*^ + *KRAS*^*G12V*^ vs. *U2AF1*^*WT*^ + *LacZ*, and *U2AF1*^*S34F*^ + *KRAS*^*G12V*^ vs. *U2AF1*^*WT*^ + *LacZ*.

### Normalized gene count comparisons:

Normalized gene counts for *U2AF1* and *KRAS* were obtained using DESeq2 for each pairwise comparison and plotted with Python.v3.7.7 and Jupyter notebook v6.3.0. Significance was calculated using Kruskal-Wallis test with Dunn’s multiple comparisons test using Python’s scikit_posthocs module.

### Differential splicing analysis on HBEC3kts:

Aligned .BAM files were filtered to remove nonstandard chromosomes with samtools v.1.13. Splicing quantification was performed with JuncBASE^32^ v.1.2 beta using the multiprocessing version with the following non-default parameters. First, run_preProcess_by_chr_step1.py was run with --preProcess_options “–unique -j /path/to/intron/coordinates -p 20. Next, disambiguate_junctions.py was run with --by_chr --majority_rules. run_preProcess_step3_by_chr.py was run with --LSF --lsf_queue --min_overhang 6 --num_processes 8 --force --nice. To identify and quantify alternative splicing events in each sample, run_getASEventReadCounts_multiSample.py was run with --sqlite_db_dir /path/to/sqlite_db_dir --jcn_seq_len 240 -p 20 --by_chr. Tables of raw and length-normalized counts of inclusion and exclusion isoforms were created using run_createAS_CountTables.py --jcn_seq_len 240 --num_processes 30. The full JuncBASE commands are in the juncbase_run.sh file provided in the GitHub.

JuncBASE count files were statistically analyzed with the compareSampleSets.py module, using the following non-default commands: --mt_correction BH --which_test t-test --delta_thresh 10.0. *U2AF1*^*WT*^*+LacZ* samples were compared with the mutant genotypes. Then, redundant splicing events were filtered out using the JuncBASE script makeNonRedundantAS.py. To compare splicing event type distributions between the genotypes, splicing events were filtered for padj < 0.25 and abs(ΔPSI) ≥ 10. We also filtered out junction-only alternative acceptor and alternative donor events, as these events have less read support than other categories. Additionally, we filtered for intron retention events that consisted of known junctions. Statistical differences between splicing event distributions were calculated using a Fisher’s exact test and row-wise Fisher’s exact test with R’s rstatix library.

### GSEA:

The log2 fold change (FC) values from each differential gene expression comparison was filtered for adjusted p-value (padj) < 0.05, and the filtered log2 FC values along with gene names were exported as a .RNK file for gene set enrichment analysis using a custom Python script in the GitHub. Gene set enrichment analysis was performed by inputting .RNK files generated from differential expression or splicing analysis into GSEAPreranked on the GSEA v.4.3.2 software^56^. The “Collapse/Remap to gene symbols” option was set to “No_Collapse” and default settings were used for the remaining options. The positive and negative GSEA output tables for each gene set were combined, and the normalized enrichment scores (NES) were filtered for FDR q-value < 0.25 and nominal p-value < 0.05. Filtered NES and the identities of their corresponding gene sets were plotted in heatmaps using Python.v3.7.7 and Jupyter notebook v6.3.0. NA values corresponding to gene sets where NES from certain pairwise comparisons did not pass the filters were replaced with 0 for plotting.

To generate the .RNK file for splicing changes, we took the absolute value of the ΔPSI values produced by compareSampleSets.py and filtered them for padj < 0.25. Unlike differential gene expression, multiple splicing events are possible for a given gene. To convert our results into a .RNK file readable by GSEA, we handled duplicate ΔPSI entries by keeping the entry with the highest abs(ΔPSI) value. The ΔPSI values and gene names were then exported as a .RNK file for GSEA.

### Stress granule protein gene set analysis:

A list of stress granule protein genes was obtained from Biancon et al.^21^. To generate a list of stress granule protein genes reported to also be differentially bound to by *U2AF1*^*S34*^ HBEC3kts, we filtered the stress granule protein gene list for genes that exhibited differential U2AF1 binding from an RNA-IP experiment performed with anti-U2AF1 generated by Palangat et al.^14^. Heatmaps of differentially expressed and spliced genes were then generated with this gene set.

### *U2AF1*^*S34F*^ and *KRAS*^*G12V*^ co-occcurrence:

Lung ADC patient sample mutational statuses was obtained from cBioPortal^57^. Overlapping studies as well as the TSP Nature, 2008 were excluded from analysis. Co-occurrence p value was obtained from cBioPortal’s Mutual Exclusivity analysis and the mutational status of patient samples were plotted with GraphPad Prism.

### *U2AF1*^*S34F*^ and smoking history splicing alteration status:

Lung ADC patient sample RNA-seq data was obtained from TCGA and smoking status for the patients was obtained from Campbell et al.^58^. Splicing alteration status was obtained by running JuncBASE to compare lung ADC against matched normal tissues. “jcn_only”, novel intron retention events, and events where more than 25% of the samples were missing data were excluded. Splicing event PSI medians and interquartile range (IQR) were computed from samples with a smoking designation from Campbell et al. A splicing event was considered altered in an individual sample if the PSI – median was more than 1.5xIQR for that event, and the ΔPSI was more than 10% from the median. P-values were calculated using a Mann-Whitney test.

## Supplementary Material

Supplement 1 **Document S1**. Figures S1–S3.

Supplement 2 **Table S1**. Excel file containing data for figures plotted with GraphPad Prism. Related to Figures 1A, 1D, 2D, 3A, 3D, 4B; Figures S2 F, S3 C–E.

Supplement 3 **Table S2**. Excel file of Log2 fold change values for HBEC3kt lines from DESeq2. Related to Figure 1C, Figure S3 A.

Supplement 4 **Table S3**. Excel file containing fluorescent imaging measurements. Related to Figures 3B–C.
