# Supplementary material for: U2AF1 S34F enhances tumorigenic potential of lung cells by exhibiting synergy with KRAS mutation and altering response to environmental stress
Source: bioRxiv. 2024 Sep 15:2024.09.11.612492. Preprint. [Version 1] doi: 10.1101/2024.09.11.612492 (PMC11419039; doi:10.1101/2024.09.11.612492)
Supplement: Supplement 1 — Document S1. Figures S1–S3. [file media-1.pdf]

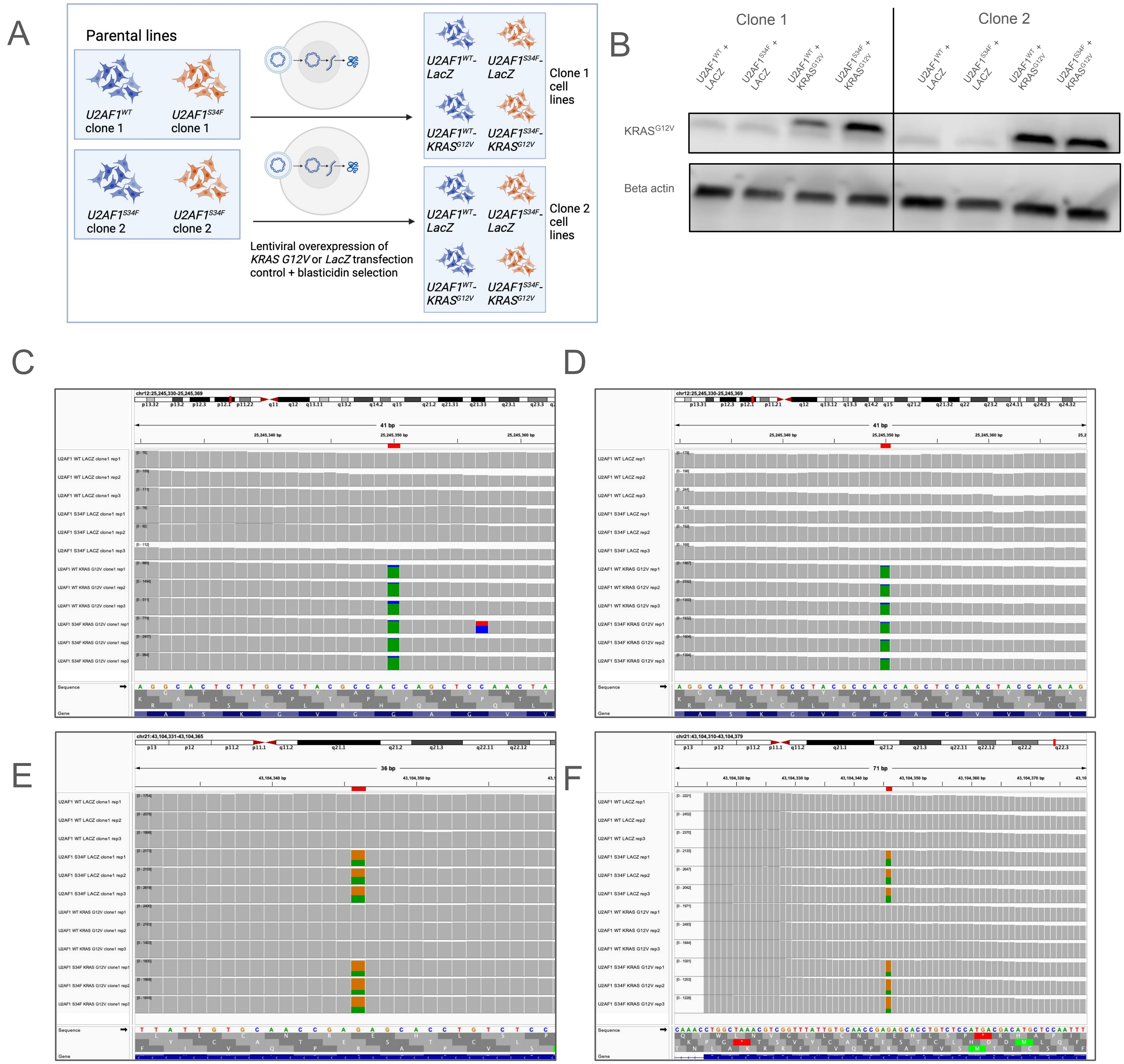

S1 Validation of cell lines for *U2AF1^{S34F}* and *KRAS^{G12V}*. (A) Schematic of cell line generation from 2 parental clones of *U2AF1^{S34F}* and *U2AF1^{WT}* HBEC3kts. **(B)** Western blot validation of *KRAS^{G12V}* in clone 1 and 2 HBEC3kts. **(C)** IGV validation of *KRAS^{G12V}* mutation for clone 1 HBEC3kts. **(D)** IGV validation of *KRAS^{G12V}* mutation for clone 2 HBEC3kts. **(E)** IGV validation of *U2AF1^{S34F}* mutation for clone 1 HBEC3kts. **(F)** IGV validation of *U2AF1^{S34F}* mutation for clone 2 HBEC3kts. The loci of *U2AF1^{S34F}* and *KRAS^{G12V}* mutations are highlighted with a red bar at the top of the IGV browser shot. Colored bars in the coverage tracks denote the presence of deviations from the reference sequence.

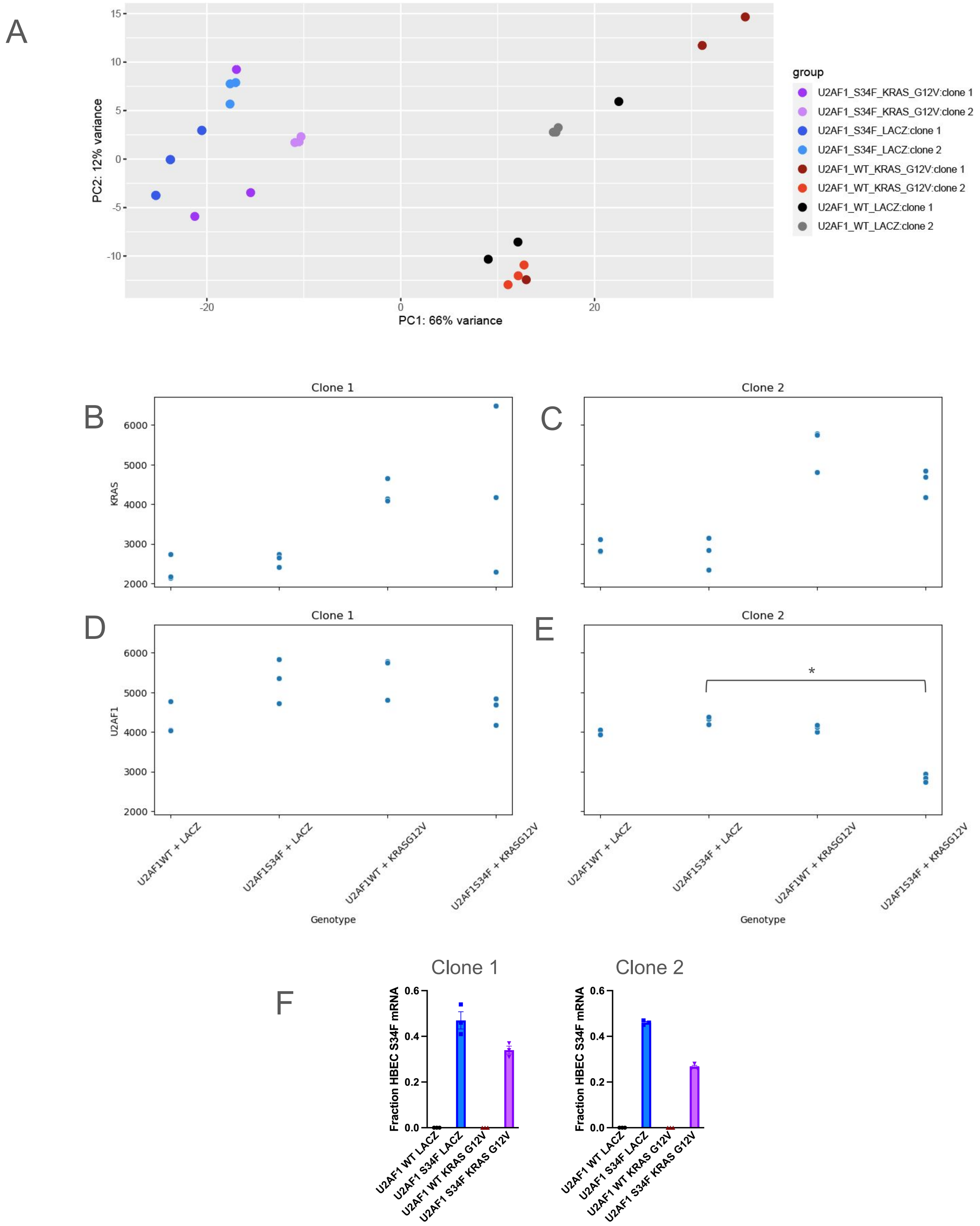

S2 Gene expression variation in *U2AF1*^{S34F} and *KRAS*^{G12V} between clones. A, PCA of clone 1 and clone 2 gene expression. **(B)** Normalized gene counts of *KRAS* in clone 1 HBEC3kts. **(C)** Normalized gene counts of *KRAS* in clone 2 HBEC3kts. **(D)** Normalized gene counts of *U2AF1* in clone 1 HBEC3kts. **(E)** Normalized gene counts of *U2AF1* in clone 2 HBEC3kts. **(F)** Fraction of *U2AF1*^{S34F} mRNA in clone 1 and clone 2 HBEC3kts. Bars represent means of S34F mRNA fraction. Error bars represent SEM. * P ≤ 0.05. See also Table S1.

A

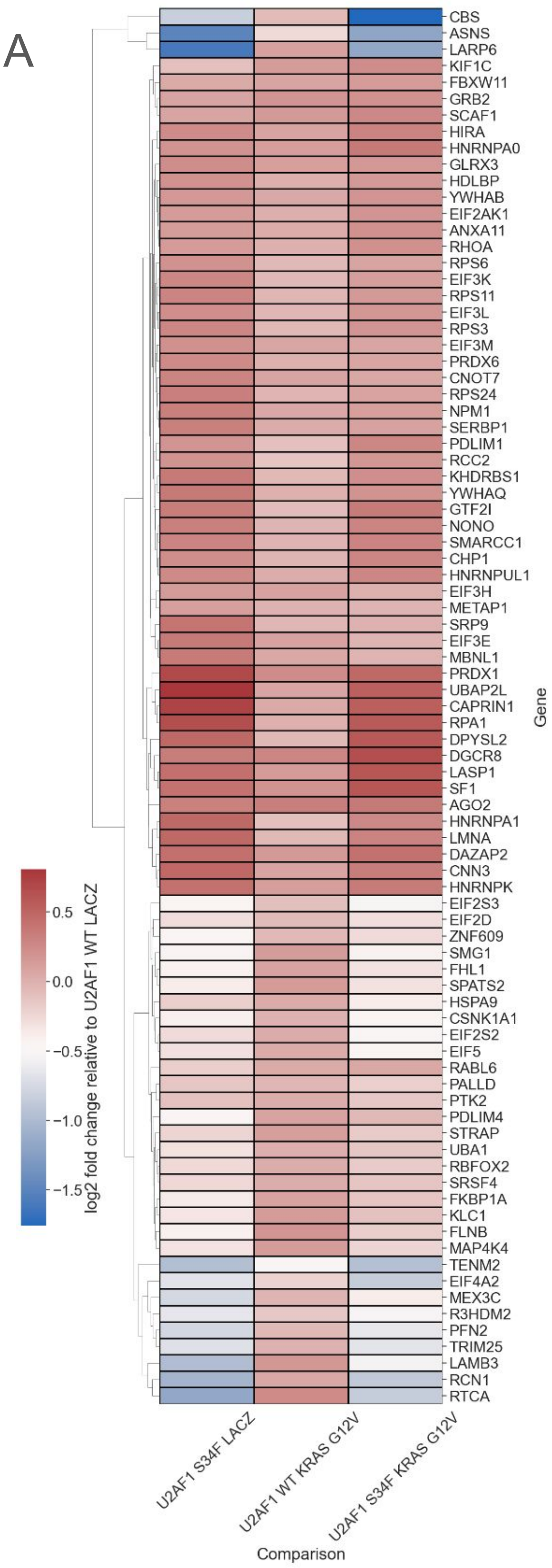

B

Genotype	Number of animals injected	Number of injection sites per animal	Number of injection sites across all animals	Total number of tumors across all animals	Tumor formation rate (%)
**U2AF1 WT LacZ clone 1**	2	2	4	0	0
**U2AF1 S34F LacZ clone 1**	2	2	4	0	0
**U2AF1 WT KRAS G12V clone 1**	2	2	4	1	25
**U2AF1 S34F KRAS G12V clone 1**	2	2	4	1	25

C

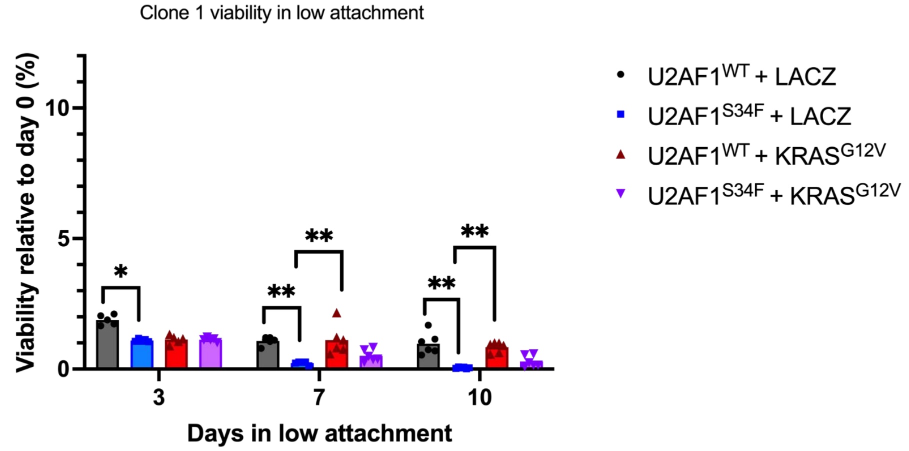

D

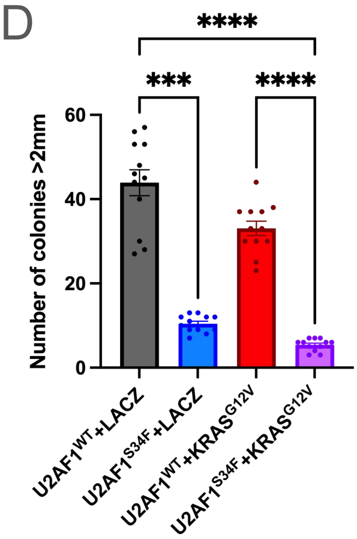

E

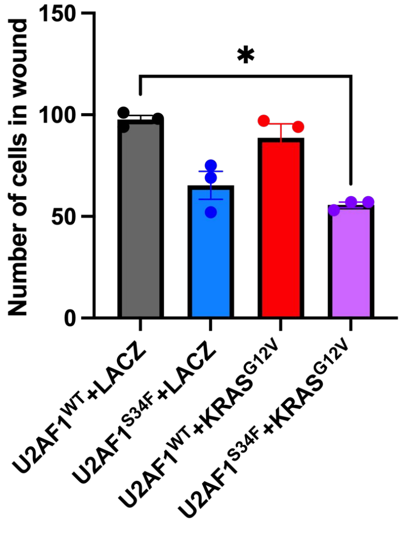

S3 Gene expression of stress granule protein genes in clone 2 transcripts with differential *U2AF1* binding and additional phenotyping of clone 1 and clone 2 HBEC3kts. (A) Heatmap of Log2FC values of stress granule protein genes with altered *U2AF1* binding and corrected p < 0.05 in clone 2 HBEC3kts. (**B)** *in vivo* tumor formation of clone 1 HBEC3kt lines. **(C)** Anchorage-independent growth in clone 1 HBEC3kt lines. Bars represent mean viability relative to day 0 in low attachment. **(D)** Clonogenicity assay of clone 2 HBEC3kts. Bars represent average numbers of colonies larger than 2mm. **(E)** Potential of clone 2 HBEC3kts to invade wound after 3 hours in 2-D culture. Bars represent average number of cells in wound. Error bars represent SEM. * P ≤ 0.05, ** P ≤ 0.01, *** P ≤ 0.001, **** P ≤ 0.0001. See also Table S1, Table S2.
